# Allogeneic Hematopoietic Stem Cell Transplantation for Adult Acute Lymphoblastic Leukemia: Results from a Single Center, 1993-2011

**Published:** 2017-01-01

**Authors:** Ipek Yonal-Hindilerden, Sevgi Kalayoglu-Besisik, Nuray Gurses-Koc, Fehmi Hindilerden, Deniz Sargin

**Affiliations:** 1 Department of Internal Medicine, Division of Hematology, Istanbul University, Istanbul Medical Faculty, Istanbul, Turkey; 2 Hematology Clinic, Istanbul Bakırkoy Sadi Konuk Training and Research Hospital, Istanbul, Turkey; 3 Department of Internal Medicine, Division of Hematology, Istanbul Medipol University, Istanbul, Turkey

**Keywords:** Acute lymphoblastic leukemia, Allogeneic hematopoietic stem cell transplantation, Overall survival, Progression-free survival

## Abstract

**Background:** For adult ALL patients, the indications and appropriate timing of allogeneic hematopoietic stem cell transplantation (AHSCT) continue to be debated. The primary aim of this single-institution study was to compare the results of our adult ALL patients that had been allografted with those reported in the current literature.

**Subjects and Methods:** This study included 53 consecutive adults with acute lymphoblastic leukemia (ALL) who underwent allogeneic hematopoietic stem cell transplantation (AHSCT) with myeloablative (92%) and reduced-intensity (8%) conditioning between 1993 and 2011.

**Results:** Mean patient age was 27 years (SD:8.62) and donor age was 33.7 years (SD:9.47). Fourteen patients were in first remission; 21 in ≥2nd remission, 15 in relapse and 3 had primary refractory leukemia. Thirty-four, 15 and 4 patients received busulfan plus cyclophosphamide, cyclophosphamide/total body irradiation and fludarabine-based regimens, respectively. For graft-versus-host disease (GVHD) prophylaxis, cyclosporine plus methotrexate were used. Forty-six donors were related and 7 were unrelated. Thirty patients received granulocyte-colony stimulating factor (G-CSF) mobilized peripheral blood and 23 received bone marrow as stem cell source. Twenty-six patients relapsed at a mean duration of 11.3 months (SD:19.1). Forty-four patients succumbed to their disease after a mean follow-up of 13.6 months (SD:19.5). The cause of mortality was relapse (n=24; 54.5%) and transplant-related etiologies (n=20; 45.5%). The estimated five year probabilities of overall survival (OS) and progression-free survival (PFS) were 37% and 12%, respectively.

**Conclusion:** By multivariate analyses, transplantation in first remission was the most important predictor of transplant success.

## Introduction

 Adult acute lymphoid leukemia (ALL) is a rare disease, for which the indications and the appropriate timing of allogeneic hematopoietic stem cell transplantation (AHSCT) continue to be discussed.^1^^-^^3^ With current intense chemotherapy protocols, majority of ALL patients younger than 55 years achieve remission but most invariablyexperience relapse.^4^ Postremission treatment strategies include prolonged chemotherapy, autologous stem cell transplantation and AHSCT. Recently, targeted therapy with monoclonal antibodies have changed the policy of ALL treatment.^5^^-^^8^ Tyrosine kinase inhibitors (TKIs) have improved long-term outcome of adult patients with Philadelphia chromosome-positive ALL.^9^^-^^12^ This study aimed to retrospectively compare the results of our adult ALL patients that had been allografted with that reported in the current literature.

## SUBJECTS AND METHODS

 This study investigated clinical course of 53 consecutive patients who underwent AHSCT for adult ALL at Istanbul University Istanbul Medical Faculty between October 2003 to January 2011. Fifty-three patients (35 male, 18 female) with a mean age of 27 years (SD 8.62) were included. At the time of AHSCT, 35 patients were in complete remission (CR) (14 CR1 and 21 ≥CR2), 15 had relapsed disease and 3 had primary refractory leukemia. Myeloablative conditioning regimens (n=49; 92%) consisted of cyclophosphamide/total body irradiation (Cys-TBI) (n=15) or busulfan**-**cyclophosphamide (Bu-Cys) (n=34), whereas for reduced-intensity conditioning, fludarabine-based regimens were used (n=4; 8%). The source of stem cells was granulocyte-colony stimulating factor (G-CSF) mobilized peripheral blood (n=30, 56.6%) and bone marrow (n=23; 43.4%). Cyclosporine with short course of methotrexate (CYA-MTX) were used for graft-versus-host disease (GVHD) prophylaxis.

## Results

 The mean donor age was 33.7 years (SD:9.47). Males comprised 54.7% (n=29) of donor population. There were 46 related (86.7%) and 7 unrelated (13.3%) donors. Pretransplant disease status and donor characteristics are summarized in Table 1. The incidence of unrelated transplantation in primary refractory group was higher as compared to other groups (p=0.004) (Table 1). In total, 26 of 53 patients (49.1%) relapsed after a mean duration of 11.3 months (SD:19.1). Fourty-four of 53 patients (83%) succumbed to their disease after a mean follow-up of 13.6 months (SD:19.5). Pretransplant status was main predictor of mortality (the rate of mortality in relapsed and primary refractory group was 100%, 64.3% in CR1 and 81% in ≥CR2; p=0.065). The cause of mortality was relapse (n=24; 54.5%) and transplant-related etiologies (n=20; 45.5%) (Table 2). Transplant-related mortality was higher in primary refractory group than the other groups (p=0.008) (Table 2). On Kruskal Walis analysis, active disease at time of transplantation was associated with a higher likelihood of shorter overall survival (OS) and progression-free survival (PFS) (p=0.001 and p=0.011, respectively).

**Figure 1:**
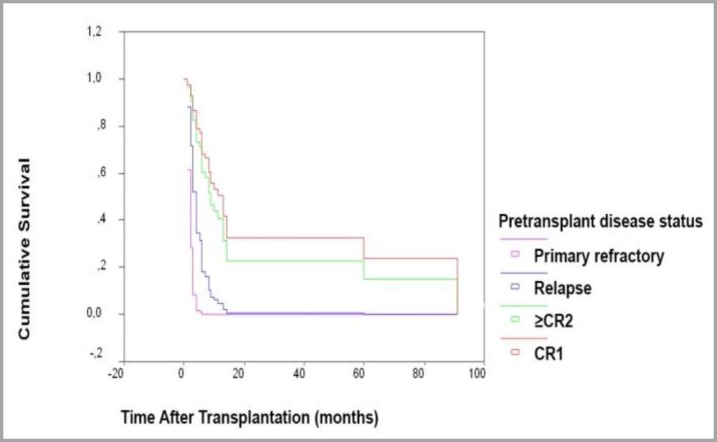
Kaplan-Meier plot. OS of ALL patients stratified by pretransplant disease status (n=53). ALL patients that underwent transplantation in CR1 predicted a higher OS compared to the other groups (OR: 2.414; 95% CI: 1.56-3.72; p<0.001).

**Figure 2:**
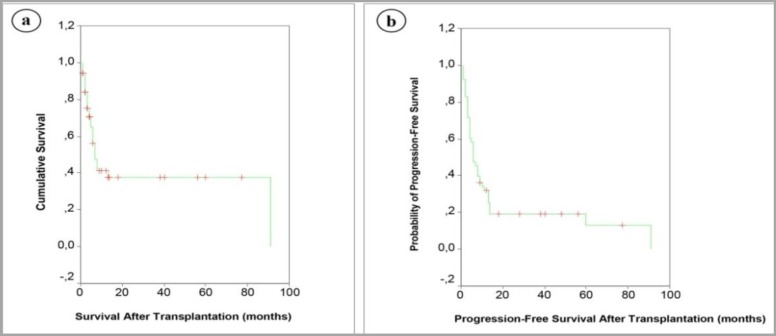
Survival outcomes and progression-free survival of ALL patients (N=53). a: Overall survival data for 53 ALL patients. OS rates were 94%, 75%, 64%, 56%, 47%, 37% and 37% at 1st, 3rd, 5th, 6th, 7th, 13th and 60th month of AHSCT, respectively. b: Progression-free survival data for 53 ALL patients. Rates of PFS were 92%, 71%, 58%, 47%, 45%, 25% and 12% at 1st, 3rd, 5th, 6th, 7th, 13th and 60th months of AHSCT, respectively.

Cox regression analysis demonstrated that the association between inferior OS and pretransplant disease status was sustained on multivariate analysis, which included age, gender, donor type (related/unrelated), source of stem cell, conditioning regimen, donor age and donor gender as covariates (OR:2.414; 95% CI:1.56-3.72; p <0.001) (Figure 1). On the other hand, prognostic significance of disease status at the time of transplantation did not maintain its significant association with PFS in multivariate analysis (p>0.01). Table 3 demonstrates that CR1 and ≥CR2 patients at the time of transplantation achieved higher OS and PFS compared to relapsed and primary refractory group (p<0.001). Mean OS and median OS for ALL patients after AHSCT was 37.42 months (95% CI, 23.48-51.36) and 7 months (95% CI, 4.86-9.14), respectively. Mean PFS and median PFS for ALL patients after AHSCT were 20.32 months (95% CI, 11.45-29.18) and 6 months (95% CI, 3.86-8.14), respectively. OS rates 1, 3, 5, 6, 7, 13 and 60 months after AHSCT were 94%, 75%, 64%, 56%, 47%, 37% and 37%, respectively (Figure 2a). Rates of PFS at 1st, 3rd, 5th, 6th, 7th, 13th and 60th month of AHSCT were 92%, 71%, 58%, 47%, 45%, 25% and 12%, respectively (Figure 2b).

**Table 1:** Characteristics of pretransplant disease status and donor types

**Pretransplant disease ** **status (N)**	**Related** **N (%)**	**Unrelated** **N (%)**
CR1 (14)	12 (85.7%)	2 (14.3%)
≥CR2 (21)	19 (90.5%)	2 (9.5%)
Relapsed disease (15)	14 (93.3%)	1 (6.7%)
Primary refractory disease (3)	1 (33.3%)	2 (66.7%)

**Table 2:** Causes of posttransplant mortality according to pretransplant disease status

**Pretransplant disease ** **status (N)**	**Transplant-related ** **mortality** **N (%)**	**Relapse-related ** **mortality** **N (%)**
CR1 (14)	3/14 (21.4%)	5/14 (35.7%)
≥CR2 (21)	8/21 (38.1%)	10/21 (47.6%)
Relapsed disease (15)	6/15 (40%)	9/15 (60%)
Primary refractory disease (3)	3/3 (100%)	0/3
Total	20/53 (37.7%)	24/53 (45. 2%)

**Table 3:** Comparison of overall survival and progression-free survival according to pretransplant disease status

**Pretransplant ** **disease status (N)**	**Posttransplant overall ** **survival (months)** **mean (SD)**	**Posttransplant ** **progression-free ** **survival (months)** **mean (SD)**
CR1 (14)	19.2 (SD:18.1)	15.2 (SD:16.9)
≥CR2 (21)	18.3 (SD:25.3)	15.6 (SD:26.1)
Relapsed disease (15)	4.4 (SD:2.5)	3.8 (SD;2.3)
Primary refractory disease (3)	1.6 (SD:1.1)	1.6 (SD:1.1)
Total	13.6 (SD:19.5)	11.3 (SD:19.1)

## Discussion

 Our study summarized an 18-year experience in 53 consecutive ALL patients treated at Istanbul University Medical Faculty, Adult Stem Cell Transplantation Unit. In total, 49.1% of patients relapsed at a mean of 11.3 months and 83% succembed to their disease after a mean period of 13.6 months. In our study, 5-year OS and PFS were 37% and 12%, respectively. In study by Doney K. et al. the rate of death was reported as 60.8% among 161 consecutive adult ALL patients who underwent AHSCT with myeloablative conditioning between 1998 and 2006.^13^ In that study, the estimated 5-year probability of OS was 38% and transplantation in CR1 was the most important predictor of successful transplantation, in line with our results. ^13^ Doney K. et al. reported a significantly lower rate of non-relapse mortality (NRM) in ALL patients who underwent AHSCT in CR1 as compared to those in ≥CR2 or in relapse (21%, 35% and 46%, respectively).^13^ Likewise, in our study, NRM in CR1, ≥CR2 and relapsed group was 21.4%, 38.1% and 40%, respectively. The most frequent cause of death in our study poulation was recurrent ALL, in line with the above-mentioned study (54.5% and 52%, respectively). In that study, 47% of patients underwent transplantation in CR1, 35% in second or greater CR and 18% in relapse, whereas in our study rates were 26.4%, 39.6% and 28.3%, respectively.^13^ Also, in our study, 5.7% of patients had primary refractory disease at time of AHSCT. Although the 5-year OS in our study group was similar to the aforementioned study,^13^ the rate of death was higher in our study (83% and 60.8%, respectively). This can be explained by the lower incidence of patients who underwent AHSCT in CR1 in our study (26.4% and 47%, respectively). Recently, Giaccone L. et al. summarized a single-center, 12-year experience of 88 consecutive patients were diagnosed with ALL at Division of Hematology at Città della Salute e della Scienza Hospital, University of Torino, Torino, Italy.^14^ In that study, 40 of 88 patients underwent AHSCT (85% in CR1 (n=34), 12.5% in CR2 (n=5) and 0.025% in progression (n=1)).^14^ Conditioning regimens were myeloablative in the majority of cases (95%). Median time from diagnosis to allografting was 6 months (range, 4-10 months). In contrast to our study, the incidence of G-CSF mobilized peripheral blood was significantly higher in that study as compared to our study (93% and 56.6%, respectively).^14^ As opposed to study by Doney et.al. and our study, Giaccone et al. reported a lower rate of death after AHSCT (60.8%, 83% and 47.5%, respectively).^13^^,^^14^ Mortality due to disease recurrence was higher in Giaccone L. et al. as compared to the study by Doney K. et.al. and the current study (84.2%, 52% and 54.5%, respectively).^13^^,^^14^

In the study by Giaccone et al. NRM was extremely low. This finding may be explained by a high incidence of early relapse (17.5% at 1 year), the high incidence of transplantation in CR1 (85%) and young median age (41 years).^14^ In the aforementioned patients undergoing transplantation, OS and PFS at 5 years were both 53%. In conclusion, OS at 5 years was higher in the study by Giaccone L. et al. as compared to study by Doney K. et.al. and the current study (53%, 38% and 37%, respectively).^13^^,^^14^ This is likely due to the higher incidence of ALL patients in CR1 before transplantation and the low median time from diagnosis to AHSCT in the study by Giaccone et al.^14^ In our study, 5-year PFS was lower compared to the study by Giaccone et al. (12% and 53%, respectively).

This can be explained by the higher incidence of active-disease before AHSCT and the lack of minimal residual disease (MRD) monitoring in our center, which represents an independent risk factor and may help to identify patients who would most benefit from AHSCT and redefining relapse.^15^^,^^16^ The optimal role of TKIs remains to be defined in transplant-eligible patients.^9^^-^^12^ Monoclonal antibodies, such as rituximab, improved survival in CD20-positive ALL; blinatumomab, resulted in OS rates of 40% to 50% in a refractory or relapsed disease and inotuzubab ozogamicin, resulted in 55% CR in a similar setting.^5^^-^^8^

Novel targeted therapies (monoclonal antibodies or TKIs) either used as a bridge to transplant or as maintenance in high risk patients or both may achieve better disease control and improve transplantation outcomes, leading to higher cure rates. In conclusion, the policy for the treatment of ALL at our institution has been considering an AHSCT in CR1 in young high-risk patients.

## CONCLUSION

 ALL patients that underwent transplantation in CR1 predicted a successful transplantation. In our study, the 5-year OS was 37 which is in line with some previously published data. In our adult ALL population, posttransplant relapse remains the major cause of morbidity and mortality. In our study, the 5-year PFS was 12%, which was lower compared to the published studies. This can be explained by the higher incidence of active-disease before AHSCT and the lack of MRD monitoring in our center, which represents an independent risk factor and may help to identify patients who would most benefit from AHSCT and to redefine relapse. Novel targeted therapies (monoclonal antibodies or TKIs) either used as a bridge to transplant or as maintenance in high risk patients or in both conditions may achieve better disesase control and improve transplantation outcomes, leading to higher cure rates.
